# Supplementary material for: Properties of wood composite plastics made from predominant Low Density Polyethylene (LDPE) plastics and their degradability in nature
Source: PLoS One. 2020 Aug 3;15(8):e0236406. doi: 10.1371/journal.pone.0236406 (PMC7398493; doi:10.1371/journal.pone.0236406)
Supplement: S1 Dataset — (DOCX) [file pone.0236406.s001.docx]

**Appendix 1. Physical and Mechanical Properties of WPC**

**Table 1. Density**

| LDPE:WF | Replications | Density (g/cm^3^) | Mean (g/cm^3^) | St dev |
| --- | --- | --- | --- | --- |
| 95:5 | 1 | 0.67 | 0.65 | 0.02 |
|  | 2 | 0.63 |  |  |
|  | 3 | 0.66 |  |  |
| 90:10 | 1 | 0.68 | 0.68 | 0.01 |
|  | 2 | 0.67 |  |  |
|  | 3 | 0.69 |  |  |
| 85:15 | 1 | 0.66 | 0.66 | 0.03 |
|  | 2 | 0.69 |  |  |
|  | 3 | 0.63 |  |  |

| **Anova (Analysis of Variance)**  **Tests of Between-Subjects Effects** | | | | | |
| --- | --- | --- | --- | --- | --- |
| Dependent Variable: Density | | | | | |
| Source | Type III Sum of Squares | df | Mean Square | F | Sig. |
| Corrected Model | .001^a^ | 2 | .001 | 1.209 | .362 |
| Intercept | 3.973 | 1 | 3.973 | 8316.372 | .000 |
| Factor_A | .001 | 2 | .001 | 1.209 | .362 |
| Error | .003 | 6 | .000 |  |  |
| Total | 3.977 | 9 |  |  |  |
| Corrected Total | .004 | 8 |  |  |  |
| a. R Squared = ,287 (Adjusted R Squared = ,050) | | | | | |

***Duncan Multiple Range Test* (DMRT)**

|  | Ratio | N | Subset |
| --- | --- | --- | --- |
|  |  |  | 1 |
| Duncan^a,,b^ | 95:5 | 3 | .6533 |
|  | 85:5 | 3 | .6600 |
|  | 90:10 | 3 | .6800 |
|  | Sig. |  | .199 |
| Alpha = ,05. | | | |

**Table 2. Moisture Content (MC)**

| LDPE:WF | Replications | MC (%) | Mean (%) | St dev |
| --- | --- | --- | --- | --- |
| 95:5 | 1 | 0.32 | 0.41 | 0.09 |
|  | 2 | 0.50 |  |  |
|  | 3 | 0.42 |  |  |
| 90:10 | 1 | 0.36 | 0.59 | 0.21 |
|  | 2 | 0.78 |  |  |
|  | 3 | 0.64 |  |  |
| 85:15 | 1 | 1.00 | 1.03 | 0.14 |
|  | 2 | 0.91 |  |  |
|  | 3 | 1.18 |  |  |

**Anova (Analysis of Variance)**

**Tests of Between-Subjects Effects**

Dependent Variable: Moisture Content

| Source | Type III Sum of Squares | df | Mean Square | F | Sig. |
| --- | --- | --- | --- | --- | --- |
| Corrected Model | .603^a^ | 2 | .302 | 12.437 | .007 |
| Intercept | 4.148 | 1 | 4.148 | 171.013 | .000 |
| Factor_A | .603 | 2 | .302 | 12.437 | .007 |
| Error | .146 | 6 | .024 |  |  |
| Total | 4.897 | 9 |  |  |  |
| Corrected Total | .749 | 8 |  |  |  |
| a. R Squared = ,806 (Adjusted R Squared = ,741) | | | | | |

***Duncan Multiple Range Test* (DMRT)**

| Duncan^a,,b^ | | | |
| --- | --- | --- | --- |
| Ratio | N | Subset | |
|  |  | 1 | 2 |
| 95:5 | 3 | 0.4133a |  |
| 90:10 | 3 | 0.5933a |  |
| 85:15 | 3 |  | 1.0300b |
| Sig. |  | 0.207 | 1.000 |

. Alpha = ,05.

**Table 3. Water Absorption (WA) 2 h**

| LDPE:WF | Replications | WA 2h (%) | Mean (%) | St dev |  |
| --- | --- | --- | --- | --- | --- |
| 95:5 | 1 | 0.25 | 0.23 | 0.025 |  |
|  | 2 | 0.20 |  |  |  |
|  | 3 | 0.23 |  |  |  |
| 90:10 | 1 | 0.55 | 0.59 | 0.089 |  |
|  | 2 | 0.69 |  |  |  |
|  | 3 | 0.53 |  |  |  |
| 85:15 | 1 | 0.12 | 0.13 | 0.012 |  |
|  | 2 | 0.13 |  |  |  |
|  | 3 | 0.15 |  |  |  |

**Anova (Analysis of Variance)**

| **Tests of Between-Subjects Effects** | | | | | |
| --- | --- | --- | --- | --- | --- |
| Dependent Variable: WA 2h | | | | | |
| Source | Type III Sum of Squares | df | Mean Square | F | Sig. |
| Corrected Model | .347^a^ | 2 | .173 | 62.829 | .000 |
| Intercept | .919 | 1 | .919 | 333.201 | .000 |
| Factor_A | .347 | 2 | .173 | 62.829 | .000 |
| Error | .017 | 6 | .003 |  |  |
| Total | 1.282 | 9 |  |  |  |
| Corrected Total | .363 | 8 |  |  |  |
| 1. R Squared = ,954 (Adjusted R Squared = ,939)   ***Duncan Multiple Range Test* (DMRT)**   \|  \| \| \| \| \| --- \| --- \| --- \| --- \| \| Duncan^a,,b^ \| \| \| \| \| Ratio \| N \| Subset \| \| \| 1 \| 2 \| \| 85:5 \| 3 \| .1350a \|  \| \| 95:5 \| 3 \| .2323a \|  \| \| 90:10 \| 3 \|  \| .5913b \| \| Sig. \|  \| .064 \| 1.000 \| \| Alpha = ,05. \| \| \| \| \|  \| \| \| \| | | | | | |

**Table 4. Water Absorption (WA) 24 h**

| LDPE:WF | Replications | WA 2h (%) | Mean (%) | St dev |  |
| --- | --- | --- | --- | --- | --- |
| 95:5 | 1 | 1.90 | 1.68 | 0.547 |  |
|  | 2 | 2.08 |  |  |  |
|  | 3 | 1.05 |  |  |  |
| 90:10 | 1 | 3.04 | 2.90 | 1.500 |  |
|  | 2 | 4.33 |  |  |  |
|  | 3 | 1.34 |  |  |  |
| 85:15 | 1 | 6.41 | 5.31 | 1.100 |  |
|  | 2 | 5.33 |  |  |  |
|  | 3 | 4.21 |  |  |  |

**Anova (Analysis of Variance)**

| **Tests of Between-Subjects Effects** | | | | | |
| --- | --- | --- | --- | --- | --- |
| Dependent Variable: WA 24h | | | | | |
| Source | Type III Sum of Squares | df | Mean Square | F | Sig. |
| Corrected Model | 20.578^a^ | 2 | 10.289 | 8.206 | .019 |
| Intercept | 97.944 | 1 | 97.944 | 78.109 | .000 |
| Factor_A | 20.578 | 2 | 10.289 | 8.206 | .019 |
| Error | 7.524 | 6 | 1.254 |  |  |
| Total | 126.046 | 9 |  |  |  |
| Corrected Total | 28.102 | 8 |  |  |  |
| 1. R Squared = ,732 (Adjusted R Squared = ,643) | | | | | |

***Duncan Multiple Range Test* (DMRT)**

|  | | | |
| --- | --- | --- | --- |
| Duncan^a,,b^ | | | |
| Ratio | N | Subset | |
|  |  | 1 | 2 |
| 95:5 | 3 | 1.6767c |  |
| 90:10 | 3 | 2.9033c |  |
| 85:5 | 3 |  | 5.3167d |
| Sig. |  | .228 | 1.000 |
| . Alpha = ,05. | | | |

**Table 5. Thickness Swelling (TS) 2h**

| LDPE:WF | Replications | TS 2h (%) | Mean (%) | | St dev |  |
| --- | --- | --- | --- | --- | --- | --- |
| 95:5 | 1 | 0.21 | 0.38 | | 0.288 |  |
|  | 2 | 0.72 |  |  |  |  |
|  | 3 | 0.23 |  |  |  |  |
| 90:10 | 1 | 1.41 | 1.33 | | 0.138 |  |
|  | 2 | 1.41 |  |  |  |  |
|  | 3 | 1.17 |  |  |  |  |
| 85:15 | 1 | 0.31 |  | |  |  |
|  | 2 | 0.60 | 0.38 | | 0.176 |  |
|  | 3 | 0.28 |  |  | |  |

**Anova (Analysis of Variance)**

| **Tests of Between-Subjects Effects** | | | | | |
| --- | --- | --- | --- | --- | --- |
| Dependent Variable: TS 2h | | | | | |
| Source | Type III Sum of Squares | df | Mean Square | F | Sig. |
| Corrected Model | 1.761^a^ | 2 | .881 | 19.733 | .002 |
| Intercept | 4.466 | 1 | 4.466 | 100.089 | .000 |
| Factor_A | 1.761 | 2 | .881 | 19.733 | .002 |
| Error | .268 | 6 | .045 |  |  |
| Total | 6.495 | 9 |  |  |  |
| Corrected Total | 2.029 | 8 |  |  |  |
| a. R Squared = ,868 (Adjusted R Squared = ,824) | | | | | |

***Duncan Multiple Range Test* (DMRT)**

|  | | | |
| --- | --- | --- | --- |
| Duncan^a,,b^ | | | |
| Ratio | N | Subset | |
|  |  | 1 | 2 |
| 95:5 | 3 | .3867a |  |
| 85:5 | 3 | .3967a |  |
| 90:10 | 3 |  | 1.3300b |
| Sig. |  | .956 | 1.000 |
| Alpha = ,05. | | | |

**Table 6. Thickness Swelling (TS) 24h**

| LDPE:WF | Replications | TS 24h (%) | Mean (%) | | St dev |  |
| --- | --- | --- | --- | --- | --- | --- |
| 95:5 | 1 | 0.97 | 1.27 | | 0.373 |  |
|  | 2 | 1.69 |  |  |  |  |
|  | 3 | 1.16 |  |  |  |  |
| 90:10 | 1 | 2.93 | 2.68 | | 1.410 |  |
|  | 2 | 3.96 |  |  |  |  |
|  | 3 | 1.17 |  |  |  |  |
| 85:15 | 1 | 7.66 |  | |  |  |
|  | 2 | 3.31 | 5.97 | | 2.333 |  |
|  | 3 | 6.95 |  |  | |  |

| **Anova (Analysis of Variance)**  **Tests of Between-Subjects Effects** | | | | | |
| --- | --- | --- | --- | --- | --- |
| Dependent Variable: TS 24h | | | | | |
| Source | Type III Sum of Squares | df | Mean Square | F | Sig. |
| Corrected Model | 34.890^a^ | 2 | 17.445 | 6.908 | .028 |
| Intercept | 98.671 | 1 | 98.671 | 39.074 | .001 |
| Factor_A | 34.890 | 2 | 17.445 | 6.908 | .028 |
| Error | 15.151 | 6 | 2.525 |  |  |
| Total | 148.712 | 9 |  |  |  |
| Corrected Total | 50.041 | 8 |  |  |  |
| 1. R Squared = ,697 (Adjusted R Squared = ,596) | | | | | |

***Duncan Multiple Range Test* (DMRT)**

| Duncan^a,,b^ | | | |
| --- | --- | --- | --- |
| Ratio | N | Subset | |
|  |  | 1 | 2 |
| 95:5 | 3 | 1.2733c |  |
| 90:10 | 3 | 2.6867c |  |
| 85:15 | 3 |  | 5.9733d |
| Sig. |  | .318 | 1.000 |
| Alpha = ,05. | | | |

**Table 7. Modulus of Elasticity (MOE)**

| LDPE:WF | Replications | MOE(kgf/cm^2^) | Mean (%) | St dev |
| --- | --- | --- | --- | --- |
| 95:5 | 1 | 20007.62 | 47664.71 | 8687.01 |
|  | 2 | 45951.66 |  |  |
|  | 3 | 40875.91 |  |  |
| 90:10 | 1 | 67558.37 | 61782.14 | 13349.07 |
|  | 2 | 71270.37 |  |  |
|  | 3 | 46517.62 |  |  |
| 85:15 | 1 | 52007.93 | 35611.73 | 13749.80 |
|  | 2 | 37662.61 |  |  |
|  | 3 | 53323.58 |  |  |

| **Anova (Analysis of Variance)**  **Tests of Between-Subjects Effects** | | | | | |
| --- | --- | --- | --- | --- | --- |
| Dependent Variable: MOE | | | | | |
| Source | Type III Sum of Squares | df | Mean Square | F | Sig. |
| Corrected Model | 4.906E10 | 2 | 2.453E10 | .975 | .430 |
| Intercept | 9.064E10 | 1 | 9.064E10 | 3.602 | .106 |
| Factor_A | 4.906E10 | 2 | 2.453E10 | .975 | .430 |
| Error | 1.510E11 | 6 | 2.516E10 |  |  |
| Total | 2.907E11 | 9 |  |  |  |
| Corrected Total | 2.000E11 | 8 |  |  |  |
| a. R Squared = ,245 (Adjusted R Squared = -,006) | | | | | |

***Duncan Multiple Range Test* (DMRT)**

|  | | |
| --- | --- | --- |
| Duncan^a,,b^ | | |
| Ratio | N | Subset |
|  |  | 1 |
| 95:5 | 3 | 35611.7300 |
| 90:10 | 3 | 61782.1033 |
| 85:5 | 3 | 203664.7067 |
| Sig. |  | .256 |
| Alpha = ,05. | | |

**Table 8. Modulus of Rupture (MOR)**

| LDPE:WF | Replications | MOR(kgf/cm^2^) | Mean (%) | St dev |
| --- | --- | --- | --- | --- |
| 95:5 | 1 | 17.02 | 22.82 | 2.89 |
|  | 2 | 18.71 |  |  |
|  | 3 | 32.73 |  |  |
| 90:10 | 1 | 22.02 | 17.34 | 3.06 |
|  | 2 | 9.51 |  |  |
|  | 3 | 20.50 |  |  |
| 85:15 | 1 | 2.77 | 4.96 | 9.61 |
|  | 2 | 4.54 |  |  |
|  | 3 | 7.59 |  |  |

**Anova (Analysis of Variance)**

| **Tests of Between-Subjects Effects** | | | | | |
| --- | --- | --- | --- | --- | --- |
| Dependent Variable: MOR | | | | | |
| Source | Type III Sum of Squares | df | Mean Square | F | Sig. |
| Corrected Model | 501.917^a^ | 2 | 250.959 | 5.932 | .038 |
| Intercept | 2036.717 | 1 | 2036.717 | 48.144 | .000 |
| Factor_A | 501.917 | 2 | 250.959 | 5.932 | .038 |
| Error | 253.826 | 6 | 42.304 |  |  |
| Total | 2792.460 | 9 |  |  |  |
| Corrected Total | 755.744 | 8 |  |  |  |
| a. R Squared = ,664 (Adjusted R Squared = ,552) | | | | | |

***Duncan Multiple Range Test* (DMRT)**

| Duncan^a,,b^ | | | |
| --- | --- | --- | --- |
| Ratio | N | Subset | |
|  |  | 1 | 2 |
| 85:15 | 3 | 4.9667b |  |
| 90:10 | 3 | 17.3433b | 17.3433a |
| 95:5 | 3 |  | 22.8200a |
| Sig. |  | .059 | .342 |
| Alpha = ,05. | | | |

**Table 9. Internal Bonding**

| LDPE:WF | | Replications | | IB(kgf/cm^2^) | Mean (%) | | St dev | |
| --- | --- | --- | --- | --- | --- | --- | --- | --- |
| 95:5 | 1 | | 0.15 | | | 0.14 | | 0.009 |
|  | 2 | | 0.14 | | |  |  |  |
|  | 3 | | 0.13 | | |  |  |  |
| 90:10 | 1 | | 0.46 | | | 0.40 | | 0.237 |
|  | 2 | | 0.61 | | |  |  |  |
|  | 3 | | 0.14 | | |  |  |  |
| 85:15 | 1 | | 0.25 | | | 0.24 | | 0.048 |
|  | 2 | | 0.14 | | |  |  |  |
|  | 3 | | 0.29 | | |  |  |  |

**Anova (Analysis of Variance)**

| **Tests of Between-Subjects Effects** | | | | | |
| --- | --- | --- | --- | --- | --- |
| Dependent Variable: IB | | | | | |
| Source | Type III Sum of Squares | df | Mean Square | F | Sig. |
| Corrected Model | .106^a^ | 2 | .053 | 2.629 | .151 |
| Intercept | .619 | 1 | .619 | 30.805 | .001 |
| Factor_A | .106 | 2 | .053 | 2.629 | .151 |
| Error | .121 | 6 | .020 |  |  |
| Total | .845 | 9 |  |  |  |
| Corrected Total | .226 | 8 |  |  |  |
| a. R Squared = ,467 (Adjusted R Squared = ,289) | | | | | |

***Duncan Multiple Range Test* (DMRT)**

| Duncan^a,,b^ | | |
| --- | --- | --- |
| Ratio | N | Subset |
|  |  | 1 |
| 95:5 | 3 | .1400 |
| 85:15 | 3 | .2433 |
| 90:0 | 3 | .4033 |
| Sig. |  | .070 |
| Alpha = ,05. | | |
