# Supplementary material for: Properties of wood composite plastics made from predominant Low Density Polyethylene (LDPE) plastics and their degradability in nature
Source: PLoS One. 2020 Aug 3;15(8):e0236406. doi: 10.1371/journal.pone.0236406 (PMC7398493; doi:10.1371/journal.pone.0236406)
Supplement: S2 Dataset — (DOCX) [file pone.0236406.s002.docx]

**Appendix 2. Degradation of WPC**

**Table 3. Weigh of WPC Before and After Grave Yard Test**

| No | | Ratio of  LDPE : WF | Replications | Initial Weight (g) | Weight after grave yard test on day (g) | | | | |
| --- | --- | --- | --- | --- | --- | --- | --- | --- | --- |
|  |  |  |  |  | 10^th^ | 20 ^th^ | 30 ^th^ | 40 ^th^ | 50 ^th^ |
| 1 | | 85 : 15 | 1  2  3 | 8.47  8.51  9.04 | 8.42  8.47  8.98 | 8.35  8.40  8.93 | 8.31  8.35  8.89 | 8.27  8.31  8.83 | 8.22  8.26  8.78 |
| 2 | | 90 : 10 | 1  2  3 | 7.68  8.19  9.20 | 7.63  8.14  9.16 | 7.58  8.07  9.11 | 7.52  8.02  9.08 | 7.48  7.97  9.04 | 7.41  7.92  8.99 |
| 3 | | 95 : 5 | 1  2  3 | 7.77  8.38  9.30 | 7.75  8.36  9.26 | 7.71  8.34  9.22 | 7.68  8.30  9.18 | 7.64  8.27  9.15 | 7.58  8.22  9.10 |
| 4 | Control  (durian wood) | | 1 | 5.41 | 4.92 | 4.37 | 3.95 | 3.47 | 3.02 |
|  |  |  | 2 | 5.39 | 5.02 | 4.01 | 3.61 | 3.34 | 3.11 |
|  |  |  | 3 | 6.19 | 5.82 | 5.11 | 4.52 | 4.07 | 2.97 |

**Anova (Analysis of Variance)**

| Source | df | Sum of Square | Mean of Square | F cal | F table |
| --- | --- | --- | --- | --- | --- |
| Treatment | 3 | 4125,80 | 1375,27 | 177,98^**^ | 0,00 |
| Error | 7 | 54,08 | 7,72 |  |  |
| Total | 11 | 84500,33 |  |  |  |

Remarks: ^**^ Statistically significant

Fcal > F table, means statistically significant to the weight loss after grave yard test

| **Between-Subjects Factors** | | | | | | | | | | | | |  |  |  |  |  |  |  |  |
| --- | --- | --- | --- | --- | --- | --- | --- | --- | --- | --- | --- | --- | --- | --- | --- | --- | --- | --- | --- | --- |
|  | | |  | | Value Label | | | | N | | | |  |  |  |  |  |  |  |  |
| Degradation Test | | | 0 | | Control | | | | 3 | | | |  |  |  |  |  |  |  |  |
|  |  |  | 1 | | WF 5% | | | | 3 | | | |  |  |  |  |  |  |  |  |
|  |  |  | 2 | | WF 10% | | | | 3 | | | |  |  |  |  |  |  |  |  |
|  |  |  | 3 | | WF 15% | | | | 3 | | | |  |  |  |  |  |  |  |  |
| **Tests of Between-Subjects Effects** | | | | | | | | | | | | | | | | | | | |  |
| Dependent Variable:Weight Loss | | | | | | | | | | |  | | | | |  |  | | |  |
| Source | | Type III Sum of Squares | | | | | Df | | | | Mean Square | | | | | F | Sig. | | |  |
| Corrected Model | | 4125.809^a^ | | | | | 3 | | | | 1375.270 | | | | | 177.983 | .000 | | |  |
| Intercept | | 79658.499 | | | | | 1 | | | | 79658.499 | | | | | 1.031E4 | .000 | | |  |
| Treatment | | 4125.809 | | | | | 3 | | | | 1375.270 | | | | | 177.983 | .000 | | |  |
| Error | | 54.089 | | | | | 7 | | | | 7.727 | | | | |  |  | | |  |
| Total | | 84500.335 | | | | | 11 | | | |  | | | | |  |  | | |  |
| **Multiple Comparisons**  Weight Loss; Tukey HSD | | | | | |  | | | |  | | | |  |  | | | |  | |
| (I) Test of Degradation | (J) Test of Degradation | | | | | Mean Difference (I-J) | | | | Std. Error | | | | Sig. | 95% Confidence Interval | | | | | |
|  |  |  |  |  |  |  |  |  |  |  |  |  |  |  | Lower Bound | | | Upper Bound | | |
| Control | WF 5% | | | | | -43.9967^*^ | | | | 2.26965 | | | | .000 | -51.5096 | | | -36.4837 | | |
|  | WF 10% | | | | | -43.1333^*^ | | | | 2.26965 | | | | .000 | -50.6463 | | | -35.6204 | | |
|  | WF 15% | | | | | -43.2200^*^ | | | | 2.53755 | | | | .000 | -51.6197 | | | -34.8203 | | |
| WF 5% | Control | | | | | 43.9967^*^ | | | | 2.26965 | | | | .000 | 36.4837 | | | 51.5096 | | |
|  | WF 10% | | | | | .8633 | | | | 2.26965 | | | | .980 | -6.6496 | | | 8.3763 | | |
|  | WF 15% | | | | | .7767 | | | | 2.53755 | | | | .989 | -7.6230 | | | 9.1764 | | |
| WF 10% | Control | | | | | 43.1333^*^ | | | | 2.26965 | | | | .000 | 35.6204 | | | 50.6463 | | |
|  | WF 5% | | | | | -.8633 | | | | 2.26965 | | | | .980 | -8.3763 | | | 6.6496 | | |
|  | WF 15% | | | | | -.0867 | | | | 2.53755 | | | | 1.000 | -8.4864 | | | 8.3130 | | |
| WF 15% | Control | | | | | 43.2200^*^ | | | | 2.53755 | | | | .000 | 34.8203 | | | 51.6197 | | |
|  | WF 5% | | | | | -.7767 | | | | 2.53755 | | | | .989 | -9.1764 | | | 7.6230 | | |
|  | WF 10% | | | | | .0867 | | | | 2.53755 | | | | 1.000 | -8.3130 | | | 8.4864 | | |
| Based on observed means. The error term is Mean Square (Error) = 7,727.  *. The mean difference is significant at the 0,05 level. | | | | | | | | | | | | | | | | | | | | |
| **Weight Loss** | | | | | | | | | | | |  |  |  |  |  |  |  |  |  |
| Tukey HSD |  | | |  | | | |  | | | |  |  |  |  |  |  |  |  |  |
| Degradation Test | N | | | Subset | | | | | | | |  |  |  |  |  |  |  |  |  |
|  |  |  |  | 1 | | | | 2 | | | |  |  |  |  |  |  |  |  |  |
| Control | 3 | | | 53.8300 | | | |  | | | |  |  |  |  |  |  |  |  |  |
| WF 10% | 3 | | |  | | | | 96.9633 | | | |  |  |  |  |  |  |  |  |  |
| WF 15% | 2 | | |  | | | | 97.0500 | | | |  |  |  |  |  |  |  |  |  |
| WF 5% | 3 | | |  | | | | 97.8267 | | | |  |  |  |  |  |  |  |  |  |
| Sig. |  | | | 1.000 | | | | .983 | | | |  |  |  |  |  |  |  |  |  |
| Means for groups in homogeneous subsets are displayed.  Based on observed means. The error term is Mean Square(Error) = 7,727. | | | | | | | | | | | | | | | | | | | | |

**Test of DTA (*Differential Thermal Analysis)***

Table 5. Results of DTA test on LDPE and WPC with various compounds

| Type of sample | LDPE (Control) | WPC 95:5 | WPC 90:10 | WPC 85:15 |
| --- | --- | --- | --- | --- |
| Temperature (°C) | 390 | 435 | 405 | 325 |


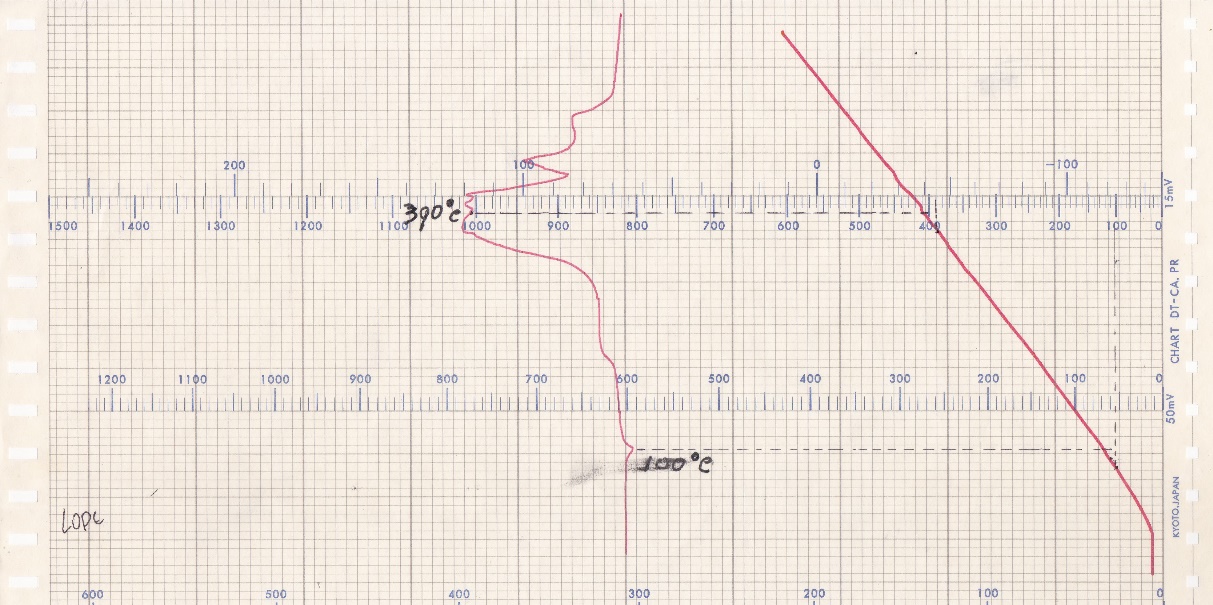


Figure i. DTA test on neat LDPE


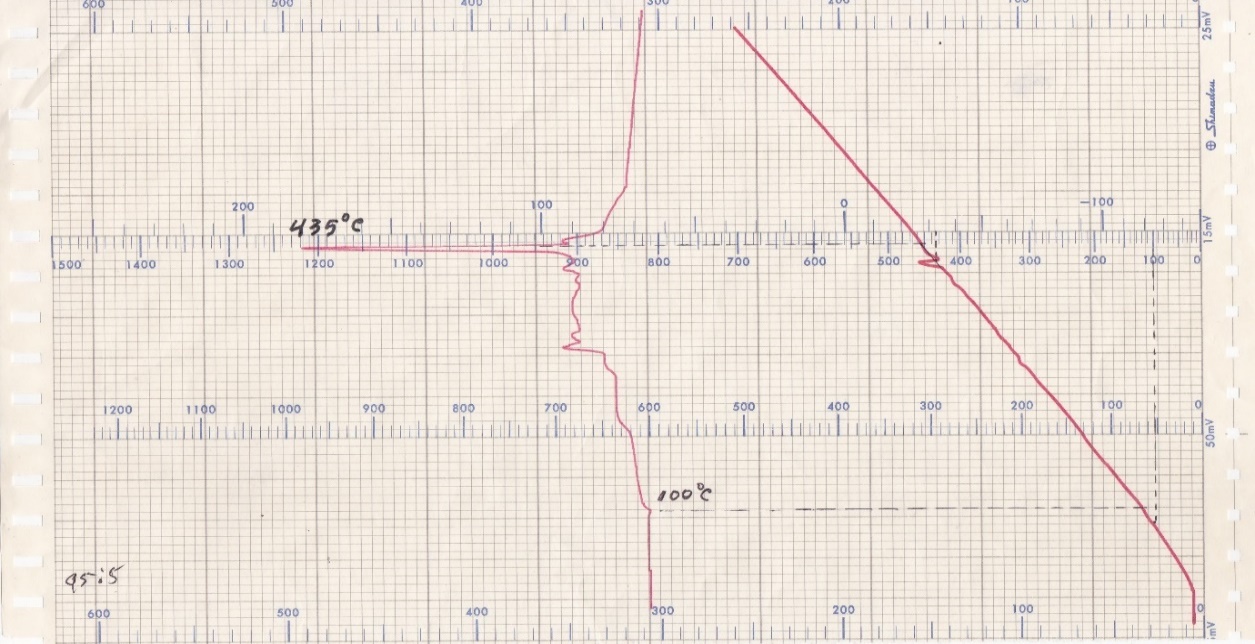


Figure ii. DTA test on WPC (95:5)


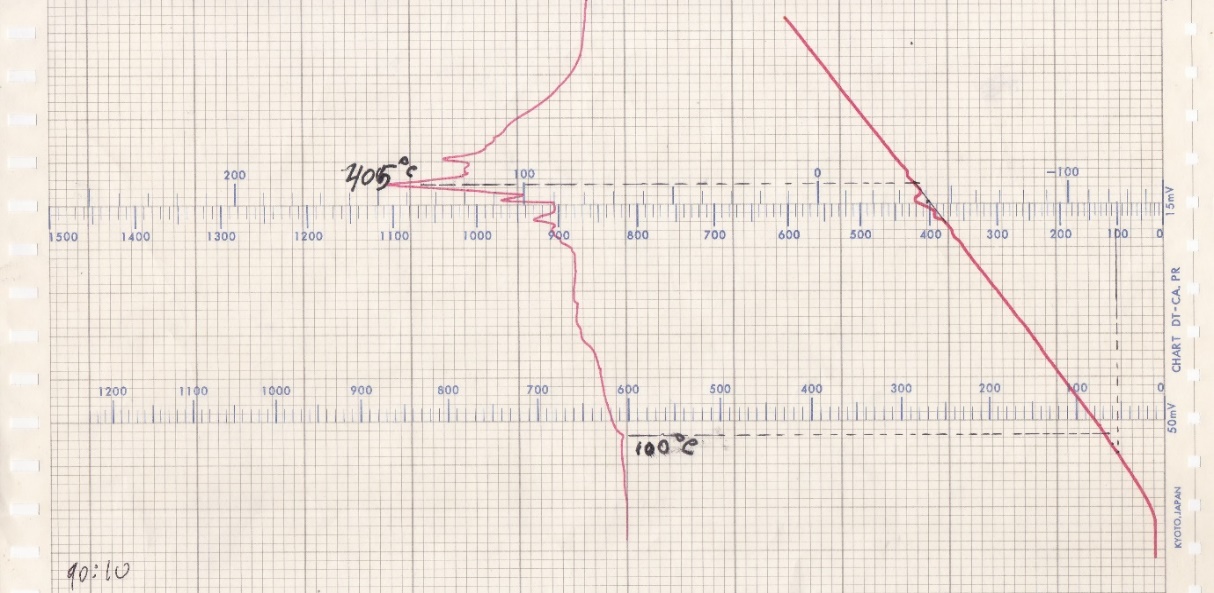


Figure iii. DTA test on WPC (90:10)


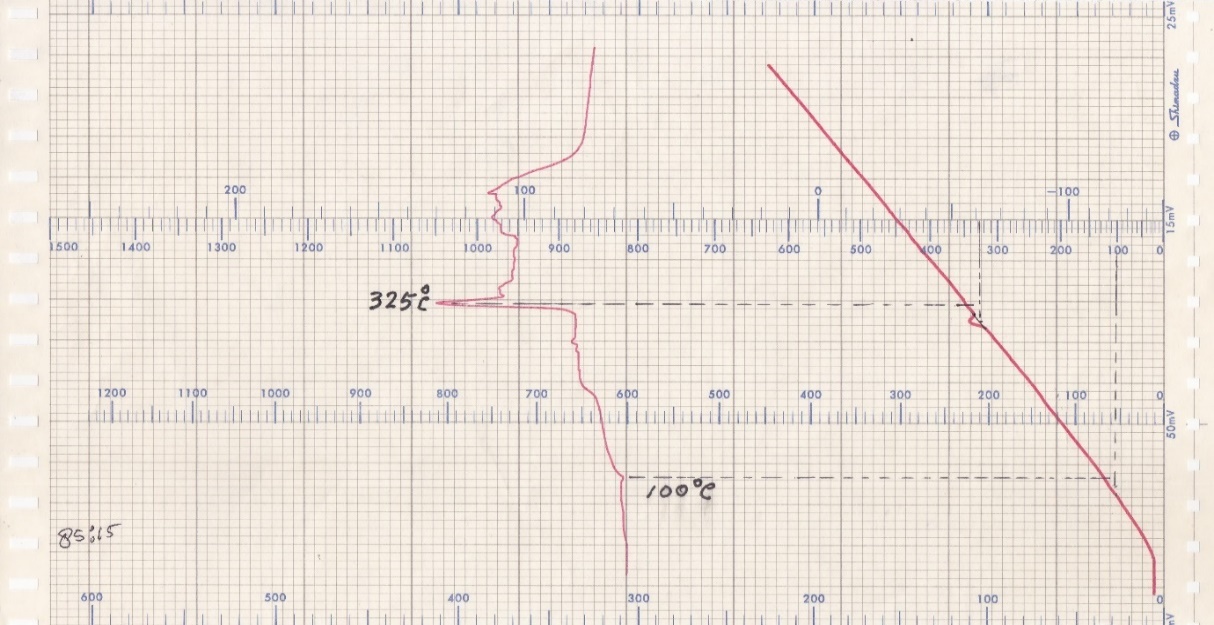


Figure iv. DTA test on WPC (85:15)

**Durability test against termite attack**

Table 6. Resistance of WPC against Termite Assay

| No. | Ratio | Weight loss (%) | Class of Resistance | Remarks |
| --- | --- | --- | --- | --- |
| 1. | 95:5 | 0.04 | I | Very durable |
| 2. | 90:10 | 0.08 | I | Very durable |
| 3. | 85:15 | 0.06 | I | Very durable |

**Anova (Analysis of Variance)**

| Source | df | SS | MS | Fcal | F table |
| --- | --- | --- | --- | --- | --- |
| Treatment | 2 | 0,003 | 0,001 | 0,731^*^ | 0,520 |
| Error | 6 | 0,012 | 0,002 |  |  |
| Total | 9 | 0,049 |  |  |  |

Remarks: ^*^ Significant

Summary: Fcal > F tab, means significant to weight loss

| **Between-Subjects Factors** | | | |
| --- | --- | --- | --- |
|  |  | Value Label | N |
| WPC Degradation | 1 | A(5%) | 3 |
|  | 2 | B(10%) | 3 |
|  | 3 | C(15%) | 3 |

| **Tests of Between-Subjects Effects** | | | | | |
| --- | --- | --- | --- | --- | --- |
| Dependent Variable:Weight loss | | |  |  |  |
| Source | Type III Sum of Squares | Df | Mean Square | F | Sig. |
| Corrected Model | .003^a^ | 2 | .001 | .731 | .520 |
| Intercept | .034 | 1 | .034 | 16.621 | .007 |
| Treatment | .003 | 2 | .001 | .731 | .520 |
| Error | .012 | 6 | .002 |  |  |
| Total | .049 | 9 |  |  |  |
| Corrected Total | .015 | 8 |  |  |  |
| a. R Squared = ,196 (Adjusted R Squared = -,072) | | | |  |  |

| **Multiple Comparisons** | | | | | | | |
| --- | --- | --- | --- | --- | --- | --- | --- |
| Weight Loss  Tukey HSD | |  |  |  |  | |  |
| (I) Uji Degradasi Papan | (J) Uji Degradasi Papan | Mean Difference (I-J) | Std. Error | Sig. | 95% Confidence Interval | | |
|  |  |  |  |  | Lower Bound | Upper Bound | |
| A(5%) | B(10%) | -.0433 | .03672 | .506 | -.1560 | .0693 | |
|  | C(15%) | -.0300 | .03672 | .707 | -.1427 | .0827 | |
| B(10%) | A(5%) | .0433 | .03672 | .506 | -.0693 | .1560 | |
|  | C(15%) | .0133 | .03672 | .931 | -.0993 | .1260 | |
| C(15%) | A(5%) | .0300 | .03672 | .707 | -.0827 | .1427 | |
|  | B(10%) | -.0133 | .03672 | .931 | -.1260 | .0993 | |
| Based on observed means.  The error term is Mean Square(Error) = ,002. | | | | |  |  | |

| **Weight Loss** | | | |  |
| --- | --- | --- | --- | --- |
| Tukey HSD | |  | |  |
| Test of WPC Degradation | N | | Subset | |
|  |  |  | 1 | |
| A (5%) | 3 | | .0367 | |
| C (15%) | 3 | | .0667 | |
| B (10%) | 3 | | .0800 | |
| Sig. |  | | .506 | |

| Means for groups in homogeneous subsets are displayed.  Based on observed means.  The error term is Mean Square(Error) = ,002. |
| --- |
