# Supplementary material for: Properties of wood composite plastics made from predominant Low Density Polyethylene (LDPE) plastics and their degradability in nature
Source: PLoS One. 2020 Aug 3;15(8):e0236406. doi: 10.1371/journal.pone.0236406 (PMC7398493; doi:10.1371/journal.pone.0236406)
Supplement: Attachment — Submitted filename: Response to Reviewers_June 22 2020.docx [file pone.0236406.s003.docx]

June 22, 2020

Professor Deniz Aydemir, PhD

Academic Editor

PLOS ONE

Department of Forest Industrial Engineering,

Faculty of Forestry, Bartin University, Turkey

Dear Professor Aydemir,

Thank you for your time and effort spent in handling our manuscript (Manuscript ID: PONE-D-19-35172) with a titled ‘Properties of Wood Composite Plastics Made from Predominant Low Density Polyethylene (LDPE) Plastics and Their Degradability in Nature’for its possible publication in the Plos One. Followings are authors’ responses to Plos One requirement and reviewers’ comments & suggestions.

**#Journal Requirements:**

When submitting your revision, we need you to address these additional requirements.

1. Please ensure that your manuscript meets PLOS ONE's style requirements, including those for file naming. The PLOSONE style templates can be found at

http://www.journals.plos.org/plosone/s/file?id=wjVg/PLOSOne_formatting_sample_main_body.pdf and<http://www.journals.plos.org/plosone/s/file?id=ba62/PLOSOne_formatting_sample_title_authors_affiliations.pdf>

Authors’ response: We have incorporated the format of manuscript according to Plos One guideline.

2. Thank you for stating the following in the Competing Interests/Financial Disclosure* (delete as necessary) section:"AN thanks to Universitas Sumatera Utara (USU) for funding this research underscheme of PenelitianTerapan TALENTA-USU year of 2018, contract number2590/UN5.1.R/PPM/2018 date of March 16, 2018."We note that one or more of the authors are employed by a commercial company: "PT Horiba Indonesia, Jl"a) Please provide an amended Funding Statement declaring this commercial affiliation, as well as a statement regardingthe Role of Funders in your study. If the funding organization did not play a role in the study design, data collection and analysis, decision to publish, or preparation of the manuscript and only provided financial support in the form of authors 'salaries and/or research materials, please review your statements relating to the author contributions, and ensure you have specifically and accurately indicated the role(s) that these authors had in your study. You can update author roles in the Author Contributions section of the online submission form. Please also include the following statement within your amended Funding Statement.“The funder provided support in the form of salaries for authors [insert relevant initials], but did not have any additional role in the study design, data collection and analysis, decision to publish, or preparation of the manuscript. The specific roles of these authors are articulated in the ‘author contributions’ section.”If your commercial affiliation did play a role in your study, please state and explain this role within your updated Funding

Statement.

Authors’ response: We have broken down the acknowledgment into authors contributions, funding statement, competing interest statement, data availability statement (supporting information), and acknowledgment both in the online form as well as in the manuscript as shown in the bottom part of the revised manuscript.

b) Please also provide an updated Competing Interests Statement declaring this commercial affiliation along with any other relevant declarations relating to employment, consultancy, patents, products in development, or marketed products, etc.

Within your Competing Interests Statement, please confirm that this commercial affiliation does not alter your adherenceto all PLOS ONE policies on sharing data and materials by including the following statement: "This does not alter ouradherence to PLOS ONE policies on sharing data and materials.” (as detailed online in our guide for authorshttp://journals.plos.org/plosone/s/competing-interests). If this adherence statement is not accurate and there arerestrictions on sharing of data and/or materials, please state these. Please note that we cannot proceed withconsideration of your article until this information has been declared.Please include both an updated Funding Statement and Competing Interests Statement in your cover letter. We willchange the online submission form on your behalf.Please know it is PLOS ONE policy for corresponding authors to declare, on behalf of all authors, all potential competinginterests for the purposes of transparency. PLOS defines a competing interest as anything that interferes with, or couldreasonably be perceived as interfering with, the full and objective presentation, peer review, editorial decision-making, orpublication of research or non-research articles submitted to one of the journals. Competing interests can be financial ornon-financial, professional, or personal. Competing interests can arise in relationship to an organization or anotherperson. Please follow this link to our website for more details on competing interests:<http://journals.plos.org/>plosone/s/competing-interests

Authors’ response: We have mentioned updated Funding Statement and Competing Interests Statement in our attached cover letter as well as in the revised manuscript.

3. Please amend either the abstract on the online submission form (via Edit Submission) or the abstract in the manuscript so that they are identical.

Authors’ response: We have altered the abstract on the online submission form with abstract in the revised manuscript.

4. Please include captions for your Supporting Information files at the end of your manuscript, and update any in-textcitations to match accordingly. Please see our Supporting Information guidelines for more information:

<http://journals.plos.org/plosone/s/supporting-information>.

Authors’ response: We have added Supporting Information (S) for explaining figures and tables in Data Availability Statement section. S1 contains information, raw data and statistical analysis of Figures 2-7 and S2 contains information, raw data and statistical analysis of Table 3, 5, and 6. Both are now available in the revised manuscript.

Reviewers' comments:

Reviewer's Responses to Questions

Comments to the Author

1. Is the manuscript technically sound, and do the data support the conclusions?The manuscript must describe a technically sound piece of scientific research with data that supports the conclusions.Experiments must have been conducted rigorously, with appropriate controls, replication, and sample sizes. Theconclusions must be drawn appropriately based on the data presented.

Reviewer #1: No

Reviewer #2: Yes

Authors’ response: We have already added the remarks of sample size of specimen testing and its replication for each examination in the manuscript. Data set in supporting information help for explaining data in the revised manuscript.

2. Has the statistical analysis been performed appropriately and rigorously?

Reviewer #1: No

Reviewer #2: Yes

Authors’ response: We have added statistical analysis on weight loss of the WPC because of both grave yard test and termite bait in line 228-229 and 288-290 (for method) and in line 401-403 and 486-488 (for results). Further, completely statistical analysis such as anova (analysis of variance) followed by DMRT (Duncan Multi Range Test) and Tukey test were available in the supporting information.

3. Have the authors made all data underlying the findings in their manuscript fully available?The PLOS Data policy requires authors to make all data underlying the findings described in their manuscript fullyavailable without restriction, with rare exception (please refer to the Data Availability Statement in the manuscript PDFfile). The data should be provided as part of the manuscript or its supporting information, or deposited to a publicrepository. For example, in addition to summary statistics, the data points behind means, medians and variance measuresshould be available. If there are restrictions on publicly sharing data—e.g. participant privacy or use of data from a third

party—those must be specified.

Reviewer #1: No

Reviewer #2: Yes

Authors’ response: We have been added supporting information containing data and statistics analysis in the additional information section.

4. Is the manuscript presented in an intelligible fashion and written in standard English?

PLOS ONE does not copyedit accepted manuscripts, so the language in submitted articles must be clear, correct, andunambiguous. Any typographical or grammatical errors should be corrected at revision, so please note any specific errorshere.

Reviewer #1: No

Reviewer #2: No

Authors’ response: The revised manuscript has been copy-editing as well as reading proof the manuscript by a native speaker who is one of the co-author of this manuscript (Dr. Deirdre MacKay). However, if this journal requires an English editing service, we would like to conduct it.

5. Review Comments to the Author

Please use the space provided to explain your answers to the questions above. You may also include additional comments for the author, including concerns about dual publication, research ethics, or publication ethics. (Please upload your review as an attachment if it exceeds 20,000 characters)

**#Reviewer1**

Reviewer #1: While variation of WF content is linear, variation of properties is not linear; this aspect is not discussed. Authors marginally discussed about extractives from wood dissolved in solvent and then dispersed in PE matrix. Therefore the final WPC material consists of PE matrix, dispersed WF particles and dispersed extractives from WPC. Actually the presence of extractives dispersed in PE is the key factor in this system, since this strongly affect the properties of final material. Therefore, a complete study should consist of four types of samples: 1) PE alone; 2) PE and extractives only (WF in xylene until complete extraction – residual WF in new xylene shows no coloration – , filtration, PE in xylene containing extractives); 3) PE and residual WF (PE and WF remained after complete extraction from sample 2;4) PE and WF (samples presented in this paper). Analysis of extractives in xylene is needed (e.g. FTIR).

Authors’ response:

Thank you for your input. Even though we have already put some discussion about linearity of the effect of variation of WF as shown in line 318-320 and 329-333 (green color text), we also added result of statistical analysis as well as the discussion of this issue as shown in lines 315-316, 325-326 (Fig. 3), 332-333, 345-346 (Fig.4), 349-350 (Fig.5), 363-365, 372-373, 380-381, 391-392 (Fig.7), 401-403 (Table 3), 405-406, 468-470, and 486-488 (Table 6).

Statement about presence of extractives in this case was only appeared in color performance part and this research was not only intended for that. Further, addition of wood filler in this case was only up to 15%. Usually, WPC was produced using wood and plastics mixture up with proportion of 30:70; 40: 60; 50:50; or *vice versa* as we described in the introduction part (green color text, line 77-99). We emphasized that WPC products with predominant plastics were fulfill the criteria of standard of physical and mechanical properties in JIS A 5908 (2003) as well as their potential degradation in the natural environment via a termite assay. However some literatures about durian wood and color performance of WPC were added (line 434-437) for making clearer as follows:

Aydemir D, Alsan M, Can A, Altuntas E, Sivrikaya H. Accelerated weathering and decay resistance of heat-treated wood reinforced polypropylene composites. DrvnaIndustrija 2019; 70(3):279-285

Chen Y, Tshabalala MA, Gao J, Stark NM, Fan Y. Color and surface chemistry changes of extracted wood flour after heating at 120°C. Wood SciTechnol 2014: 48:137-150.

Chen Y, Stark NM, Tshabalala MA, Gao J, Fan Y. Weathering characteristics of wood plastic composites reinforced with extracted or delignified wood flour. Materials 2016; 9:610.

Handayani S, Pangestuti EK, Dharma NA. Preservation of durian timber and coconut timber with soursop leaf extract. MATEC Web of Conferences 2019: 258: 01022

Careful language check is absolutely needed.

- Materials: sieved sawdust is wood particulates, not wood fibers.

Authors’ response: We added terminology of “wood powder” to make clearer of wood flour (WF) in text in line 130. WF is an abbreviation of wood flour not wood fibers.

- Dissolution/re-precipitation technique: Dissolution temperature (115°C) is close to boiling point of xylene (144°C); how was evaporation of xylene avoided, in order to maintain the 1:20 ratio? What was the mixing time after addition of WF?

Authors’ response: Ratio of 1:20 (LDPE: xylene) has been prepared prior to heating. After 30 minutes heating and the temperature increased in the range 105-115°C (melting point of LDPE); LDPE started to melt and dissolve in the xylene. When all the LDPE was dissolved, wood flour (WF) was added. Stirring was done into the mixture up to homogeneous for approximately an hour. This remark was added in the text in line 148.

How was the liquid mixture transformed into pellets?

Authors’ response: Liquid mixture then was poured on the aluminum plate and placed in acid chamber. During evaporation of xylene solvent in acid chamber, the mixture was then manually cut using scissors for converting it into pellets.

- Composite processing: Weight of 15 kg is for entire surface of the board? This is much lower than 30 kgf/cm^2^ for hotpress. Why is cold press used after hot press?

Authors’ response: Yes, we used 15 kg loading for conditioning or cold press just for keeping the shape of back and face of WPC after hot press. After hot press, the WPC was still mushy and pasty in the steel-mould. In order to solidifying or hardening, conditioning in room temperature was needed. Therefore cold pressing with lower loading than hot press was applied.

- Physical properties: How many samples have been tested in order to perform statistical analysis?

Authors’ response: We used three replication for each treatment as shown in Supporting Information section (S1)

Moisture content depends on the storage conditions, since just after preparing moisture content is virtually zero. Therefore, without describing storage conditions, moisture content is meaningless. Considering the strong hydrophobicity of PE, major component, swelling, if any, might occur only at the margins of the specimen, therefore dimensional change will manifest mainly at the margins, and less inside; was this considered?

Authors’ response: Yes, we agree. Lower moisture content of WPC is because of hydrophobicity of predominant plastics. However, increasing amount of wood filler made the moisture content was linear and statistically different when amount of wood filler was 15%.

Thickness of specimens was not mentioned.

Authors’ response: We have added the thickness of specimens in the text.

Figure 1: Why 90:10 mixture had highest density? Agglomeration of WF, as described by authors, is expected since no compatibilizer was used. Final material has low homogeneity thus large variation in properties, which can induce misleading interpretation of results, and made them unsuitable for practical purpose.

Authors’ response: Even though WPC with ratio 90:10 has highest density but statistically it was not different than WPC with ratio 95: 5 and 85: 15. Inhomogeneity in the WPC system was indeed inevitable even using initiator and or compatibilizer. In this case, without compatibilizer and predominant plastics, a product of WPC has been produced and intended to reduce plastics in the environment.

What means a, b, c, d in Figure 2 and Figure 3?

Authors’ response: Different letter upper the bar (a, b, c, d) denotes that they are statistically not significant according to the Duncan Multi Range Test (DMRT) at α=0.05.

Why 90:10 mixture had highest swelling and water absorption at 2 hours since it also had highest density?

Authors’ response: Actually, standard testing for thickness swelling is 24 hours after immersing the sample into water. In this case, authors showed even though in the early stage immersion of WPC in water (2 hours) has different tendency, but in the final stage linearity has become manifest.

- Mechanical testing: Size of specimens or standard used is not mentioned.

Authors’ response: Both specimens size and standard used have been added in lines 191-192 and 202-203 in the text.

Without using PE alone as references, discussion on addition of WF is meaningless.

Authors’ response: We have put a discussion about LDPE alone as a control in a part of thermal experiment of DTA test (blue color in line 468-470 and green color in line 473-481). For others experiment such as physical, mechanical, and biodegradation we intended not to include neat LDPE because physically, mechanically, and biologically LDPE was thermoplastics and very difficult to degrade in nature.

Replace OF with OR in “Bending strength of modulus of rupture”. Which parameter is “MOE strength”? Should be either MOE, either strength.

Authors’ response: We have been replaced “of” into “or” in text line 193.

Delete “dimensions” from were determined in “mmdimensions”.

Authors’ response: We have deleted “dimensions” in the text line 195.

Please introduce values of MOE and MOR for neat LDPE in Figures 5 and 6.

Authors’ response: MOE and MOR values of neat LDPE were added in the text in line 361-362 and in line 373, respectively.

What type of device has beenused for internal bond (IB) testing? Please specify if bending tests were performed on vertical or horizontal position of thesamples.

Authors’ response: For IB testing, we also used Tensilon Universal Testing Machine (UTM). Bending tests were performed on horizontal position of the samples. These remarks have been added in the text in lines 204.and 191-193, respectively.

- Degradation: Without describing the exposure condition (e.g. sample size/mass, soil temperature, humidity, pH, graveyard test is meaningless).

Authors’ response: Exposure condition temperature range was 30.21-31.68°C, air humidity range was 68.82-70.21%, and pH range was 6.85-7.63. This data is now in the text in line 218-219.

Since adding WF could increase degradability of plastics, PE alone should be considered as reference, for all tests, as in Figure 9.

Authors’ response: We did not consider neat LDPE as reference for grave yard test since plastics including neat LDPE required very long time to deteriorate.

How many samples have been buried in the soil for degradation testing?

Authors’ response: There were 3 (three) samples for each WPC for degradation testing. Therefore the total number of sample was 9 (nine) and the data was available in Supporting Information.

What means the values in brackets in Table 3?

Authors’ response: Value in parentheses was standard deviation. Now, this remark has been available in the text under the Table 3.

Why the 90:10 mixture had highest mass loss?

Authors’ response: Even though the WPC of 90:10 has had highest mass loss, statistically it was not significantly different than those of WPC of 95:5 and WPC of 85:15 based on Tukey test at α=0.05. This remark has been added under Table 3.

Please provide a reference forequation 8 and upgrade the reference Hunter Lab. 2008 (is just direct link to Hunter Lab webpage) – e.g.

<https://support.hunterlab.com/hc/en-us/article_attachments/201439655/an07_96a.pdf>

Authors’ response: We have provided the reference for Equation 8 above the equation (green text) and the reference was upgraded as reviewer’ suggestion.

Table 4: It is not clear what is the reference sample in color analysis. Change in colour could be only an effect of leachingthe extractives into soil, not of degradation of WF itself.

Authors’ response: We did not use a reference such as neat LDPE in color analysis because neat LDPE need very long time to degrade in nature (in the soil by grave yard test). Our analysis, change in color within WPC presumably because of weathering factors such as relative humidity (RH) in the soil and lignin degradation of wood filler from the WPC (line 426-437).

From all information that DTA analysis can provide, it is not clear. What is the meaning of temperature in Table 5. The values are by far over the alteration of crystalline structure of PE or melting. They are in the range of thermal degradation, which is a complex process, especially in the case of WF, thereforecareful interpretation is needed. DTA curves should be given.

Authors’ response: Table 5 informed that addition of wood filler 5% and 10% made WPC products were more resistant to high temperature compare to either neat LDPE or WPC products with addition of 15% wood filler. This information is now in the text. DTA curves have been provided in the Supporting Information (S2).

Figure 11: Normally, preparation of WPC should assure that xylene solvent is totally removed from the system.

Authors’ response: Yes, we have been evaporated xylene solvent overnight within the pellets prior to making WPC for assuring that xylene solvent was emitted.

Replace ENCAPSULATED with INCORPORATED in “…termites haveno choice in feed sources except from wood sawdust particles encapsulated in the matrix plastics in WPC systems.

Authors’ response: We have been replaced “encapsulated” with “incorporated” in line 259.

Authors mentioned “acclimatization to the termite was conducted for 10 days after collecting termites from the nativehabitat.”, but how long were the termites in contact with WPC samples?

Authors’ response: Termites have been contact with WPC for a week and this information has been included in the text in line 271.

**#Reviewer2**

Reviewer #2: Dear Editor,

The paper is related to “Properties of Wood Composite Plastics Made from Predominant Low Density Polyethylene (LDPE) Plastics and Their Degradability in Nature”. The paper fits within the scope of the journal. I think the contributionof the data in the article to the research community will be limited. The study has to go under major language sub editing.Work can be published after language editing.

Best regards.

Assistant Professor Dr.Timucin BARDAK

Bartin University

Furniture and Decoration Program, Bartin Vocational School,

74000, Bartin - Turkey

E-mail: [timucinb@bartin.edu.tr](mailto:timucinb@bartin.edu.tr)

Authors’ response: Thank you for your comments. The manuscript has been copy-edited by a native speaker who one of the co-author (Dr. Deirdre McKay).We would like to use professional language editing service if the journal suggests or requires it.

Best regards,

Arif Nuryawan, Ph.D
